# Roles of Epigenetics and Glial Cells in Drug-Induced Autism Spectrum Disorder

**DOI:** 10.3390/biom14040437

**Published:** 2024-04-03

**Authors:** Antonei B. Csoka, Nacer El Kouhen, Samia Bennani, Bruk Getachew, Michael Aschner, Yousef Tizabi

**Affiliations:** 1 Department of Anatomy, Howard University College of Medicine, Washington, DC 20059, USA; 2 Faculty of Medicine and Pharmacy of Casablanca, Hassan II University, Casablanca 20100, Morocco; 3 Department of Pharmacology, Howard University College of Medicine, Washington, DC 20059, USA; 4 Department of Molecular Pharmacology, Albert Einstein College of Medicine, Bronx, NY 10461, USA

**Keywords:** autism spectrum disorder, epigenetics, teratogen, valproic acid, propionic acid, acetaminophen, gliosis, neuroinflammation

## Abstract

Autism spectrum disorder (ASD) is a neurodevelopmental disorder characterized by severe deficits in social communication and interaction, repetitive movements, abnormal focusing on objects, or activity that can significantly affect the quality of life of the afflicted. Neuronal and glial cells have been implicated. It has a genetic component but can also be triggered by environmental factors or drugs. For example, prenatal exposure to valproic acid or acetaminophen, or ingestion of propionic acid, can increase the risk of ASD. Recently, epigenetic influences on ASD have come to the forefront of investigations on the etiology, prevention, and treatment of this disorder. Epigenetics refers to DNA modifications that alter gene expression without making any changes to the DNA sequence. Although an increasing number of pharmaceuticals and environmental chemicals are being implicated in the etiology of ASD, here, we specifically focus on the molecular influences of the abovementioned chemicals on epigenetic alterations in neuronal and glial cells and their potential connection to ASD. We conclude that a better understanding of these phenomena can lead to more effective interventions in ASD.

## 1. Introduction

Autism spectrum disorder (ASD) is multifactorial and complex neurodevelopmental disorder that occurs in all ethnic, racial, and socioeconomic groups. According to the latest Autism and Developmental Disabilities Monitoring (ADDM) report, the prevalence of ASD has been increasing over the years, such that currently 1 in 36 eight-year-old children in the US are diagnosed with the condition. Males outnumber females at a ratio of 4:1 [1]. The diagnosis of ASD in the DSM V requires the child to meet a panel of criteria such as persistent deficits in each of three areas of social interaction and communication: social–emotional reciprocity, processing of relationships, and nonverbal communicative behaviors, plus at least two of four types of restricted repetitive patterns of behaviors and interests [1]. In 1970’s, Folstein and Rutter first suggested a genetic component to ASD based on twin studies [2,3,4]. Since then, a number of genes have been identified (discussed below). However, it is important to note that, in this case, the genetic predisposition does not necessarily translate into the phenotype without a triggering stimulus [5]. Thus, ASD may be considered a multifactorial disorder with influences such as oxidative stress, neurotransmitter imbalance, immune system dysfunction, exposure to xenobiotics, maternal infections, teratogenic infections, cholesterol metabolism, exposure to heavy metals, and some drugs affecting the outcome [6,7,8]. 

Several different classes of pharmaceutical drugs, such as selective serotonin reuptake inhibitor (SSRI) antidepressants [9], acetaminophen (APAP) (Tylenol) [10], asthma medications [11], valproic acid (VPA) [12], and opioids [13], have been implicated in the development of ASD. Additionally, various environmental compounds, such as endocrine-disrupting chemicals, including bisphenol A (BPA) [14], phthalates [15], and propionic acid (PPA, an endogenous metabolite but also used in the manufacture of herbicides) [16], may also increase the chance of ASD. PPA is frequently used in the development animal models of ASD.

For these reasons, it has been suggested that the multifactorial condition of ASD may be dependent on epigenetic effects [17,18]. But what, precisely, is meant by epigenetics?

Epigenetics is a term that refers to DNA modifications that alter gene expression without making any changes to the genetic code. The full extent of epigenetics in modulating gene expression, especially in relation to neurological diseases, is gradually being revealed. Although, as stated above, an increasing number of chemicals are being implicated in ASD, in this review we focus on examples of prenatal exposure to VPA and APAP and postnatal exposure to PPA. Both neuronal and glial contributions are also considered. Following a brief discussion of the genetics of ASD, the epigenetic effects of the abovementioned drugs on phenotypic expression, as well as potential use of this knowledge in developing novel interventions, are discussed.

## 2. ASD–Genetics

On the basis of family studies, ASD is recognized as the most heritable neurodevelopmental disorder. Since the monozygotic twin study by Barley et al. (1970), which found a concordance of autism between 60 and 90% in monozygotic twins and 5 and 40% in dizygotic twins, the heritability and contribution of genetics to ASD has been verified [4,19,20,21]. A large population-based study conducted recently on more than 2 million individuals and 680,000 families across five countries (Denmark, Finland, Sweden, Israel, and Western Australia), estimated the heritability for ASD to be 80% [22]. Also, Butler and colleagues compiled approximately 800 genes linked to ASD, arranged them in alphabetical order, and included high-resolution human chromosome ideograms to facilitate the visualization of the specific location and arrangement of ASD-associated genes [23].

In reality, ASD comprises a group of developmental behavioral disorders that are genetically heterogenous and are associated with impairments in social skills (particularly in communication) and manifest stereotypic, rigid, or repetitive behaviors. Novel analyses of gene–protein interactions and molecular function have identified at least three pathways, such as chromatin modeling, Notch, and Wnt, that affect dendritic spine profiles and neuronal growth. Approximately 50% of individuals with ASD are diagnosed with chromosome deletions/duplications (e.g., 15q11.2, BP1-BP2, 16p11.2, and 15q13.3), identified syndromes (e.g., Williams, Phelan–McDermid, and Shprintzen velocardiofacial), or single gene disorders resulting in behavioral and psychiatric conditions [23]. Hence, chromosomal microarrays may be applied with high diagnostic yield [23,24]. In addition, pharmacogenetics testing may be used to guide the selection of medications in ASD, a technique which is also used in Down syndrome, Fragile X syndrome (FMR1 gene), and the PTEN gene mutation, which encodes a phosphatase associated with extreme macrocephaly [25].

### 2.1. Epigenetics

Epigenetics, as mentioned above, is a term that refers to DNA modifications that alter gene expression without any changes to the genetic sequence [26]. It is a process that regulates gene expression through modifications of DNA bases and changes to DNA packaging in response to environmental factors and behavioral conditions [26]. Epigenetic changes are potentially reversible but the factors regulating reversibility are not yet fully understood. Through epigenetics, genes can be fully silenced, underexpressed, or overexpressed [26]. In addition, epigenetics projects environmental exposure to disease development via modification of gene expression. It is a process that is present throughout an organism’s lifetime, with potential transmission to the next generation [27]. Epigenetic changes may be brought about by numerous factors such as maternal care, breastfeeding, physical activity or inactivity, mitochondrial dysfunction, hyperglycemia, menopause, and aging [27]. Previously, we hypothesized that pharmaceuticals [28] or drugs of abuse [29] may influence the epigenetics state and have detected DNA methylation changes in a human cell line in response to the widely used antidepressant, citalopram [30]. The epigenetics state may also be influenced by integrative medicine [31]. Moreover, epigenetics may contribute to the development of metabolic diseases such as diabetes [32,33].

DNA, with its phosphate groups, is negatively charged and packaged around a positively charged histone protein octamer that contains two copies of histone proteins H2A, H2B, H3, and H4. DNA loops twice around the histone octamer forming the functional unit of DNA, the nucleoprotein called the nucleosome [34,35,36]. DNA is, thus, packaged in a “beads-on-a-string” pattern. H1 is the last histone protein that binds to the nucleosome and linker DNA, thereby stabilizing the chromatin fiber. Linker DNA is double-stranded DNA situated in between two nucleosome cores that, in association with histone H1, holds the cores together. It was recently revealed that histone H1 binding to nucleosome arrays depends on the linker DNA length and trajectory [37]. Thus, linker DNA is considered the string in the “beads-on-a-string” model on the chromatin. Chromatin is the condensed form in which DNA exists to fit in the nucleus. The aggregation of chromatin results in the formation of chromosomes. In its loose shape, chromatin is transcriptionally active and referred to as euchromatin, as opposed to the highly condensed, transcriptionally inactive state called heterochromatin [35].

There are currently three well-understood factors regulating epigenetic expression:

1. Modifications to histones that either make the chromatin available (euchromatin state) or unavailable (heterochromatin) for transcriptional processes [26,38,39]. In this context, three different mechanisms have been described. First, is histone methylation that usually silences DNA expression. Second is histone acetylation that relaxes DNA coiling, increasing its transcription. Third is the reverse process, histone deacetylation that removes an acetyl group and further tightens DNA coiling, thus decreasing gene expression.
2. DNA methylation [40,41] is a reversible mechanism whereby methyl groups (–CH3) are delivered to cytosines positioned in CpG (5′-cytosine-phosphate-guanosine-3’) nucleotides turning these cytosines into 5-methyl cytosines (5mC) [42]. When methylation occurs in cytosine-phosphate-guanine (CpG) islands in the gene promoter, interactions between the DNA and transcription factors are reduced, and the gene is silenced [4,43]. In neural cells, either hypermethylation or hypomethylation of DNA can affect learning or memory. Indeed, dysregulated methylation has been linked to neurodevelopmental disorders such as ASD [42].
3. Gene silencing may also occur via noncoding RNA (ncRNA), referring to RNA sequences that are transcribed but not translated, hence not leading to protein synthesis [43]. Because of the high proportion of ncRNA’s noncoding transcripts (more than 89%), ncRNAs were considered junk RNA; however, recent studies emphasize their crucial role in modulating the expression of the genome [44].

### 2.2. Epigenetics–ASD

The importance of epigenetic modulation in ASD is underscored by experimental models designed to study the impact of environmental exposure in a genetically predisposed subject [45]. Epigenetic changes via DNA methylation may impact several physiological and pathological processes, such as transcriptional regulation, chromosomal stability, tissue-specific gene regulation, and genome imprinting [4,43]. Abnormal DNA methylation patterns have been observed in various neurodegenerative and neurodevelopmental disorders, such as Alzheimer’s disease (AD), depression, ADHD, and ASD.

The complexity of epigenetic mechanisms renders identification of the actual process in ASD challenging [20]. For example, Angelman syndrome (AS) is a neurodevelopmental imprinting disorder characterized by severe intellectual disability, cognitive disability, and lack of speech that has a high comorbidity with autism, thus considered nowadays a syndromic form of ASD [46,47,48,49]. In 3% to 5% of cases, AS is directly caused by a DNA methylation defect in the maternal SNRPN promoter silencing the UBE3A gene [48]. Thus, AS may be considered an epigenetic disorder. The phenotypic overlap between ASD and AS suggests a possible implication of the UBE3A gene in ASD. Indeed, imprinting of this gene was one of the first identified parent-of-origin effects (referring to phenotype inheritance from the mother or father) in ASD. This provided evidence on how epigenetic mechanisms via DNA methylation can be responsible for the phenotypic manifestations in ASD [47,48,50].

### 2.3. Glial Cells–ASD

Although most studies on ASD have focused on pathological changes in neurons, including accelerated neuronal differentiation, increased cell proliferation, reduced neuronal synchronous and spontaneous activity, and impaired synaptic development, an increasing amount of research indicates that glial cells may also be of both pathological and therapeutic potential. Earlier reports, using stereological techniques, estimated glia-to-neuron ratios of 10:1 [51,52]. However, more recent reports, using the newly validated isotropic fractionator for the reliable quantification of glia and neurons, indicate that the proportion is closer to the 3:1 (glia/neuron) [53].

These cells perform pivotal functions, such as providing energetic support for neurons [54], regulation of neurotransmitters [55,56,57], formation of the blood–brain barrier (BBB) [58,59], detoxification [60,61], development and remodeling of synapses [62,63,64], control of fluid/electrolyte homeostasis [65], neuroendocrine function [66], innate immunity response [67,68], control of metabolism [69,70], and myelination [71,72]. Thus, glial cells play a key role in maintaining homeostasis, disruption of which can lead to neurodevelopmental, neuropsychiatric, and neurodegenerative diseases [72,73,74,75,76,77]. In the central nervous system (CNS), four major subsets of glial cells (microglia, astrocytes, oligodendrocytes, and synantocytes, or NG2 cells) have been identified. A brief description of each with relevance to ASD is discussed below.

Microglia, constituting 10–15% of all CNS cells, act in the innate immune response, and play a key role in neuroinflammation. They are considered the resident macrophages of the CNS, with a vital role in maintaining homeostasis due to their ability to remove debris, regulate neurogenesis, participate in the formation and elimination of neuronal synapses, and control the number of neuronal precursor cells [78,79,80,81].

As resident immune cells, microglia can polarize into different proinflammatory (M1) or anti-inflammatory (M2) phenotypes. M1 microglia activation leads to BBB dysfunction and vascular “leakage”, whereas M2 microglia act as protectors of the BBB. Under physiological conditions, microglia readily and continuously monitor the CNS microenvironment to quickly remove debris and provide repair at the damaged area. However, their overactivation is the primary cause of neuroinflammation and cellular death [78,80,82].

Recently, it has been proposed that microglial abnormalities may cause many pathological phenotypes in ASD. Specifically, single-cell data indicate differences in gene expression patterns between adult and fetal microglia, confirming an important role for microglia in CNS development. Indeed, it is believed that many ASD phenotypes are due to immune cell abnormalities, particularly, microglia. That damage to microglia may affect synaptic pruning and lead to social behavioral deficits has been confirmed in animal models. Thus, further exploration of the mechanisms resulting in microglial abnormalities in ASD may offer novel avenues in the prevention and/or development of effective therapeutics for ASD [82,83].

Astroglia, or astrocytes, are star-shaped cells that make up between 17 and 61% of the cells in the human brain, depending on the region, and like microglia exhibit heterogeneous phenotypes in response to various insults, a process known as astrocyte reactivity [82]. Astrocytes perform a myriad of essential functions, including maintenance and accuracy of brain signaling, recycling of neurotransmitters, maintenance of the BBB, modulation of ionic environment, providing metabolic support for the neurons, and regulating sphingolipid and cholesterol metabolism, where disruption of the latter has been directly linked to ASD [84,85,86].

As alluded to earlier, risk factors for ASD include genetic predispositions, disturbed brain homeostasis, and inflammation during the prenatal period that could be caused by exposure to bacteria or viruses. Maternal immune system activation is another risk factor, as it was shown that gestational inflammation in mice at embryonic day 13 or 15 results in profound ASD symptoms, likely due to the fact that during this period the neural tube has closed and progenitor cells are proliferating and migrating [87]. Many studies suggest glial involvement in the pathology of autism, as evidenced by increased expression of glia-associated proteins in the brain. It is hypothesized that alterations of astroglial function lead to a disruption in the homeostasis of the excitatory/inhibitory balance due to aberrant Ca^2+^ signaling and the eventual manifestation of ASD [87,88]. In summary, the disruption of astrocyte function may affect proper neurotransmitter metabolism, synaptogenesis, and inflammation, leading to ASD. Thus, astroglia may represent presumptive targets for novel therapeutic strategies [87,88,89].

Oligodendrocytes (ODs), the myelinating cells of the brain, represent about 75% of all glial cells in the adult CNS. In addition to axonal myelination, ODs control extracellular potassium concentration, provide metabolic and trophic supplies to myelin, secrete glial and brain-derived neurotrophic factors (GDNF and BDNF), and modulate axonal growth [90,91], all of which highlight their importance in the functioning of the CNS. The importance of ODs in the pathogenesis of neurodevelopmental and neurodegenerative disease has been verified [92]. It is of relevance to note that in the peripheral nervous system, neuroglia that are equivalent to ODs are referred to as Schwann cells [93].

Recent comparative genomic analyses of the causative genes of ASD in animal models have demonstrated that ODs may also contribute to the molecular mechanisms underlying social functioning, disturbance of which can lead to ASD [94]. Specifically, it was demonstrated that OD-lineage cells and myelination are altered in a murine model of ASD induced by the prenatal exposure to VPA [95]. ODs’ importance in neurodegenerative diseases, in general, and Parkinson’s disease, in particular, has lately been documented [92]. Moreover, ODs also express toll-like receptors (TLRs), considered of significant importance in myelin formation [71,96,97].

NG2 cells are a subset of CNS glial cells, commonly referred to as synantocytes, neuron glial 2, or nerve/glial antigen 2 (NG2). These cells display a variety of features including (i) a complex stellate morphology; (ii) an almost uniform distribution in both white and gray matter; (iii) the ability to maintain proliferation in the adult brain; (iv) ability to give rise to astrocytes and neurons to be recruited to areas of a lesion; and (v) a tendency to be intimately associated with neuronal cell bodies and dendrites [90,98,99].

NG2 cells were once considered to function solely as progenitors for oligodendrocyte ODs. However, now they are believed to have many other important functions, the dysfunction of which can lead to pathologies such as demyelinating, neurovascular disruption, neuroinflammation, and neurodegeneration [100,101,102,103,104]. They have been proposed as targets in various neurological diseases due to their ability to receive synapses from neurons and affect neuronal plasticity [105].

A link between NG2 cells and ASD is suggested by several studies. ASD and severe macrocephaly are associated with germline mutations in the *PTEN* gene. This mutation occurs in 7–27% of patients with ASD and macrocephaly and may account for up to 5% of all ASD cases as macrocephaly is found in approximately 20% of the ASD population. Indeed, it is recommended that screening for *PTEN* mutation be carried out in all cases of ASD where the head circumference is greater than 3 standard deviations above the mean for age- and sex-matched controls [106]. PTEN is best recognized for dephosphorylating phosphatidyl-inositol (3,4,5)-triphosphate and inhibiting the PI3K/AKT/mTOR signaling pathway. The *Pten^m3m4^* mouse model exhibited increased NG2 cell proliferation and accumulation of glia, with behavioral abnormalities like some individuals with ASD [107,108,109].

Now that we have described the relevant cell populations, we turn our attention to specific chemical exposures that have been implicated in the etiology of ASD.

## 3. Valproic Acid (VPA)

VPA, 2-propyl-pentanoic acid, a broad-spectrum antiepileptic drug (AED), is used to treat many types of seizures. Despite its recognized role as an AED, it is also now used to treat bipolar disorder and is approved for migraine prophylaxis and neuropathic pain. Its antiepileptic effect is mainly explained by its inhibitory action on the neuron, as follows: it increases the GABA concentration within the neuron by inhibiting GABA transaminase (GABA-T) and blocking the voltage-gated sodium channels. The pharmacodynamic effects of VPA are numerous and complex and range from diminishing the fast and transient inward Na^+^ currents, thus interfering with the mechanism of sustained and prolonged firing to a direct action on the neuron, reducing synaptic localization of glutamate-receptor subunits [110,111].

There is also evidence suggesting that VPA affects the enzymatic activity of the brain to exert a neuroprotective, anti-inflammatory, and antioxidant effect in patients suffering from epilepsy and X-linked adrenoleukodystrophy [112]. Recently, it was shown that VPA exerts an inhibitory effect on histone deacetylation. This, together with its ability to block the voltage-gated Na^+^ and Ca^++^ channels, both of which are considered tumor markers, has prompted the suggestion of its use in breast, prostate, and other types of cancers [113].

### 3.1. VPA Action in Glial Cells

In vivo and in vitro studies have confirmed VPA modulation of glial cell function. Thus, it has been shown that VPA selectively induces caspase 3-mediated apoptosis in rodent microglial cells. Although VPA does not induce apoptosis in microglia derived from adult human brains, it does decrease the expression of the microglial markers and dramatically reduces microglial phagocytosis [114]. VPA also alters gliogenesis and may result in an abnormal number of glial cells, hence, increasing the risk of neurodevelopmental disorders [115]. It has been suggested that teratogenic actions of VPA may be mediated through changes in astrocyte generation [116]. VPA affects neuronal fate and microglial function via enhancing autophagic flux in mice after traumatic brain injury [117]. VPA exposure decreases the neurogenic potential of outer radial glia in human brain organoids [118]. Indeed, because long-term VPA-induced microgliosis could be the result of altered microglia–astroglia crosstalk, it has been proposed that targeting this crosstalk could offer a novel intervention in neuroinflammatory mechanism in ASD [119].

### 3.2. VPA and Pregnancy

VPA is a well-known teratogen. Reports suggesting its teratogenicity go back to as early as the 1980s in children whose mothers took VPA in the first trimester of pregnancy. The first documented effect was an increased rate of spina bifida (neural tube defect), whereas now the congenital malformations caused by VPA encompass all the major congenital anomalies, including neural tube and heart defects, skeletal and limb abnormalities, cleft lip and palate, anomalies of the urinary tract, and cranio-facial dysmorphism [12,120,121]. In addition to these physical anomalies, VPA may also affect the cognitive function and social behavior of the offspring, leading to an ASD-like syndrome [120,121]. Numerous animal studies in rodents and nonhuman primates also confirm the teratogenic effects of VPA and its link to ASD [122,123]. Indeed, prenatal VPA exposure in rodents has been established as a reliable translational model to study the [122,123] pathophysiology of ASD [124]. In rare cases, in utero fetal exposure of VPA can cause the human fetal valproate spectrum disorder (FVSD), a disease that causes morphological defects with a distinct facial dysmorphism, congenital anomalies, and behavioral alterations that affect language development and communication. Therefore, exposure to VPA in the first trimester of pregnancy is directly related to ASD [125].

### 3.3. VPA and ASD

VPA alters the developmental patterns of the embryo. Numerous studies suggest VPA involvement in the development of ASD in neonates [124,126]. In a rat study, it was shown that VPA exposure even during the early postnatal period may precipitate ASD-like behavior [127]. A population-based investigation in Denmark indicated that the use of VPA in pregnancy raised the chances of autism in neonates by 4.42 and ASD, or Asperger’s syndrome, by 8.9% [12]. As VPA is responsible for cognitive and behavioral alterations, numerous VPA rodent models of ASD have been studied. Rodent models of ASD developed by prenatal VPA exposure confirm behavioral abnormalities like those observed in ASD. More recently, a VPA model of ASD using domestic chicks has been proposed [128].

The pathophysiology of VPA-induced ASD is not yet fully known. Nonetheless, numerous hypotheses have been presented. For example, it has been suggested that VPA’s effect on histone deacetylase (HDAC) leads to synaptic dysfunction, abnormal signaling pathways, and deficits in neurogenesis, all of which can cause ASD. HDAC refers to a class of enzymes that eliminate acetyl groups from histones and allow the histones to enclose the DNA more tightly, causing transient hyperacetylation, which can lead to transcription inhibition and an increase in apoptotic cells, culminating in reduced cell proliferation in certain brain areas. Thus, it is believed that VPA impairment of histone acetylation of the ALDH1A1 gene (an epigenetic modification) results in the downregulation of the RA-RARα pathway, responsible for autism-like synaptic and behavioral deficits [123].

On the other hand, murine autism models have shown that VPA, by disrupting the AMPK/SIRT1/PGC1α signaling pathway, causes a dysfunction in the amygdala’s interneurons accompanied by elevated ROS and caspase 3, neuroinflammation, and eventual manifestation of ASD [129]. In another murine model, it was shown that VPA upregulates miR134-5p, similar to what is observed in ASD. There has also been the suggestion that VPA disruption of the Ca^2+^/calmodulin-dependent protein kinase (CaMKII), protein kinase C (PKC), and protein kinase A (PKA) pathways in the hippocampus is responsible for the elevated risk of autism and cognitive impairments [130]. Moreover, low-dose VPA during pregnancy may result in neocortical dysgenesis due to increased neuronal projection in specific cortical layers, resulting in abnormal social behavior, cognitive changes, heightened pain sensitivity, and impaired locomotion, resembling autism symptoms [130].

In sum, the biological anomalies tying prenatal VPA to ASD include changes in metabolites affecting mitochondrial function [131], cholesterol deficiency [86], and epigenetic dysregulation of autism-linked genes, as well as in the developing cerebellum [132].

## 4. Acetaminophen (APAP)

Acetaminophen, also called N-acetyl para-aminophenol (APAP), or paracetamol, is a widely used nonopioid analgesic and antipyretic drug. APAP acts on multiple biological cascades, including (1) increasing the expression of the anti-apoptotic protein Bcl2 and decreasing the pro-apoptotic protein caspase 3; (2) reducing prostaglandin formation via competitive inhibition of the prostaglandin H2 synthase; (3) interfering with cannabinoid receptor signaling through its metabolite N-arachidonoyl-phentolamine; and (4) a metabolite, N-acetyl-p-benzo-quinone-imine, depleting glutathione in the CNS [133,134,135].

### 4.1. APAP Action in Glial Cells

APAP possesses both antioxidant and anti-inflammatory effects, at least some of which are believed to be mediated through its interaction with glial cells, in general, and microglia, in particular. Thus, in an inflammatory mouse model in which microglia are overactivated, APAP administration restored microglial function and reversed the cognitive impairment [136]. In another transgenic mouse model of AD, administration of the APAP derivative N,N′-diacetyl-p-phenylenediamine restored microglial phagocytosis and improved cognitive defects [137]. APAP also attenuated LPS-induced cognitive impairment through antioxidant and anti-inflammatory properties [138], as well as its ability to inhibit the mitochondrial permeability transition (MPT) pore and apoptotic pathway [136]. These properties are potentially responsible for the effectiveness of APAP in reducing the risk of amyotrophic lateral sclerosis [139]. However, long-term APAP treatment impaired cognitive function and BDNF in adult rat brains [140].

### 4.2. APAP and Pregnancy

APAP is the most frequently used FDA-approved over-the-counter (OTC) analgesic and antipyretic drug. It is used by the general population, including pregnant and breastfeeding mothers, for its safety profile. In fact, APAP has been widely prescribed during pregnancy for the symptomatic treatment of moderate to severe pain, fever, and headaches, because it is considered a relatively safe drug within the recommended dosage range in different countries. However, it was classified as a class B drug in pregnancy drug categories due to concerns about its safety profile during pregnancy, which prompted the FDA to relabel APAP packaging. While its hepatotoxic effects are well established, its impacts on fetal brain development during the prenatal and postnatal periods are still to be fully elucidated.

In recent years, numerous studies have questioned the safety of this drug, especially during the prenatal and early postnatal periods. It has been reported that APAP might be harmful in the embryological phase, as it disrupts hormonal balance and interferes with sex and thyroid hormone metabolism [141]. These hormones are essential for neurogenesis, neural cell differentiation and migration, synaptogenesis, and the myelination process. Indeed, it is a well-established fact that thyroid hormone deficiency severely affects brain maturation, causing intellectual disability, intellectual deficits, and neurobehavioral impairments [139,141]. In summary, the potential neurotoxic effects of APAP on the developing brain in the prenatal and postnatal periods are concerns.

### 4.3. APAP and ASD

Various studies have addressed the biological effects of APAP on the developing brain. In vivo animal studies suggest a causal relationship between prenatal APAP exposure and altered behaviors in the offspring, such as stereotyped behavior and hyperactivity [142,143]. These effects have been attributed to APAP’s reduction of BDNF levels in the striatum of rats [144,145], as well as alterations in dopamine metabolism [142,143]. Moreover, APAP may also cause oxidative stress and mitochondrial dysfunction, which can contribute to behavioral abnormalities [135].

An increasing number of human observational and animal studies [10,146,147,148] suggest that prenatal exposure to APAP is associated with an increased risk of ASD in offspring. This association is four times higher among boys [146,147]. Several animal studies also confirm the propensity to develop ASD-like symptoms, especially in male offspring following prenatal APAP exposure [146,147,148,149,150]. Prenatal APAP exposure has been associated with impairments in motor milestones, gross motor function, autistic-like symptoms of communication, externalizing behavior, internalizing behavior, sociability, and emotionality [135,146,147,148].

Timing and duration of APAP exposure are two important factors to consider when studying its relationship to neurodevelopmental impairments. In 2018, a systematic review by Bauer et al. focused on the adverse neurodevelopmental outcomes following APAP exposure. It was determined that exposure during the second trimester of gestation, as well as exposure for more than 28 days, is responsible for a significantly higher risk of ASD, ADHD, and intellectual disability (lower IQ) [146,148]. This study suggested that the longer the exposure, the higher the risk. A more recent study has verified that early postnatal exposure to APAP may also increase the risk of ASD in males [16,151]. Dosing is also critical in this context, as at a higher dosage, not only the neurons but also the glial cells would be exposed to higher oxidative stress [135].

It may, thus, be concluded that prenatal exposure to acetaminophen increases the risk of ASD particularly in genetically susceptible offspring.

## 5. Propionic Acid

Propionic acid (PPA), also known as propanoic acid, is a ubiquitous short-chain fatty acid (SCFA) present in many processed foods, as well as animal feedstocks. Biological processes regularly produce PPA or its conjugate base, propionate, through propionic coenzyme A, as well as from cholesterol oxidation [152]. PPA is also a metabolic product of enteric bacteria that can cross into the CNS and accumulate in the cells, leading to intracellular acidification [153]. PPA exerts a myriad of effects, including disturbance of several neurotransmitters’ functions. It is now well recognized that an imbalance between the excitatory and inhibitory neurotransmitters, namely, glutamate and GABA, plays a crucial role in ASD pathology. This is further supported by the high percentage of comorbidity between epilepsy and ASD [154]. PPA also affects cell proliferation and differentiation and is considered a major neuroinflammatory mediator, as it induces gliosis and neuro-inflammation through the modulation of PTEN/AKT, a pathway intimately involved in ASD [155]. These and other neurobiological effects of PPA, including mitochondrial damage, have established it as a suitable inducer of experimental models for ASD [16,152,153,154,155,156,157,158].

### 5.1. PPA–Glial Cells–ASD

At the nuclear level, PPA stimulates tumor necrosis factor alpha (TNF-α), a pro-inflammatory cytokine, gene expression and transcription, causing uncontrolled inflammation and gliosis, both of which have been implicated in ASD development [152]. Moreover, behavioral and associated morphological changes induced by PPA in the hippocampus and amygdala, brain regions that are associated with the regulation of social behavior and cognition, validate the PPA rodent model of ASD [154,158]. Specifically, morphological analyses of PPA-treated rat brains (hippocampus and amygdala) revealed reduced neuron numbers and increased numbers of glial cells, particularly those of astrocytes and microglia. Moreover, pericapillary glia were most affected, and axons were moderately demyelinated following PPA treatment [158].

### 5.2. Epigenetic Mechanisms in ASD

**Figure 1:**
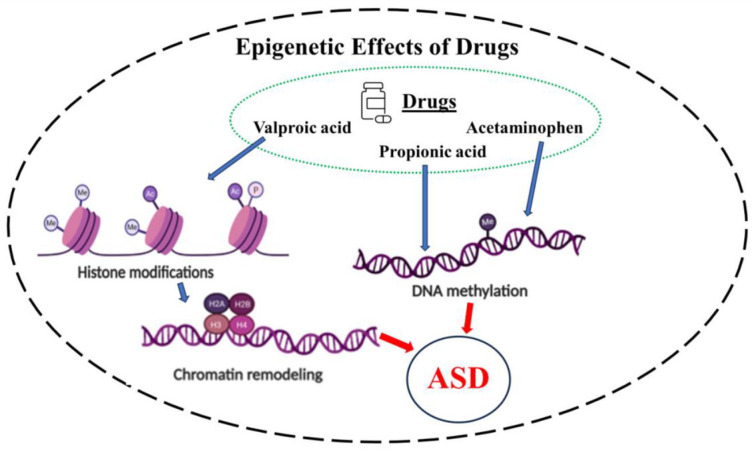
Schematic diagram depicting the direct influence of the drugs on epigenetics, leading to ASD. The two prominent epigenetic mechanisms, which affect gene expression without changing the genetic sequence, involve histone deacetylation, which results in chromatin remodeling, or DNA methylation. Interestingly, VPA may exert its epigenetic effects via histone deacetylation, whereas acetaminophen and propionic acid affect DNA methylation. Regardless of the epigenetic mechanism, however, the drug may increase the chance of ASD development [160].

As discussed above, the role of epigenetics in ASD had been suggested long ago [47]. However, the exact epigenetic mechanisms that are involved in ASD pathophysiology are still to be fully elucidated [49]. The current understanding is that the epigenetic mechanisms implicated in neurological or neurodevelopmental disorders primarily involve DNA methylation and/or histone deacetylation [159] (Figure 1).

Regarding DNA methylation, several signatures and patterns are found in specific genomic regions impacting gene expression related to synaptic function, neuronal signaling, and brain development and maturation. Hence, methylation of newly discovered genes, like the oxytocin receptor (OXTR), methyl-CpG-binding protein 2 (MECP2), Reelin (RELN), and BDNF, have been linked to ASD pathophysiology [161,162,163,164].

Specifically, MECP2 loss of function in neural cell lineages is the main cause of Rett syndrome, a neurodevelopmental disorder, mainly diagnosed in girls, which manifests with neurobehavioral, social, and communication symptoms similar to those found in patients suffering from ASD. The clinical evolution of Rett syndrome (i.e., severe neurological and neurobehavioral regressions, often one year after reaching the corresponding motor and verbal milestones) has suggested a probable inculpation of environmental factors and epigenetics in the development of this neurological disorder. The clinical correlation between these two neurodevelopmental entities has led researchers to further investigate the MECP2 gene as a genomic marker of ASD. It is well known that abnormal MECP2 expression leads to ASD in humans and ASD-like symptoms in animal models. Epigenetic manipulation of this gene can, in fact, cause abnormal behaviors like those seen in ASD. A cohort study of patients with ASD revealed significantly increased levels of MECP2 methylation compared to controls in specific CpG islands [164]. Moreover, locus-specific methylation at the MECP2 promoter was positively correlated with the development of ASD-like symptoms such as increased repetitive behaviors, anxiety, and impaired social interactions [165]. Interestingly, targeting of the MECP2 gene in the hippocampus was sufficient to provoke neurobehavioral ASD-like symptoms, suggesting a potential therapeutic benefit in targeting this gene product [166].

Additionally, recent findings have implicated oxytocin and oxytocin receptors (OXTRs) in ASD pathophysiology. Oxytocin is a neuropeptide that is extremely important during birth, mother–child bonding, and emotional functioning, as well as the development of social behaviors [161,167]. OXTR methylation can influence oxytocin’s function in social interactions [168]. Similarly, genetic alterations of RELN may also contribute to the ASD phenotype [162,163,169].

The histone deacetylation (HDAC) process is another major epigenetic mechanism that has been linked to ASD. This HDAC effect is manifested via modulation of genomic regions implicated in synaptic function, brain development, and maturation. For instance, the FMR1 gene has been associated with fragile X syndrome, a genetic condition that presents with ASD-like symptoms. A decrease in FMR1 transcription is associated with loss of the FMR1 protein that is necessary for brain maturation. HDAC, in this case, decreases the amount of chromatin available for transcription at the FMR1 promoter and may contribute to ASD manifestation [170,171].

Therefore, HDAC inhibitors may offer promising therapeutic interventions in transcriptional activators of the FMR1 gene in such conditions as fragile X syndrome and ASD [168]. Indeed, as discussed below, the utility of HDAC inhibitors as a novel therapeutic intervention in ASD has been proposed.

### 5.3. Epigenetics–Glial Cells–ASD

**Figure 2:**
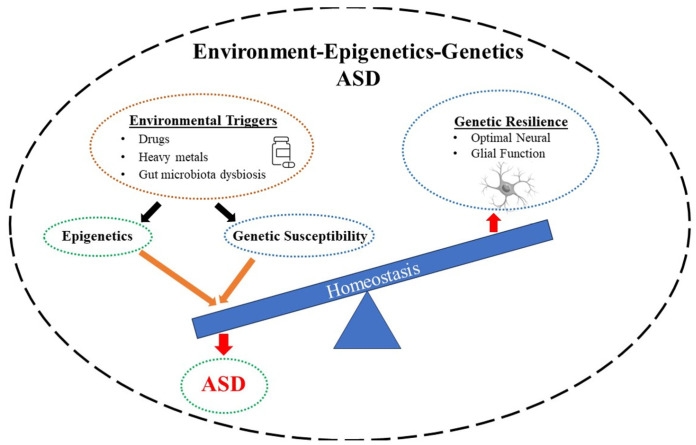
Schematic diagram depicting how environmental triggers, such as drugs, heavy metals, or dysbiosis in the gut microbiota, may result in autism spectrum disorder (ASD). This can be due to the individual’s genetic predisposition or may be brought about indirectly via epigenetic effects. The outcome is a disturbance in the homeostasis, which can also involve glial dysregulation.

It is important to note that epigenetic modulations may also extend to glial cells. That neuroinflammation may contribute to lifelong neurological disabilities including ASD has been amply verified [172]. Astrocytes and microglia, as mentioned earlier, play pivotal roles in neurodevelopment and predisposition to neurological diseases. It is now believed that a pathological glial–neuronal interplay increases the risk of ASD. Thus, it is hypothesized that fetal neuroinflammation, as well as parental stress, via gene–environment interactions (i.e., epigenetic mechanism), reprogram glial phenotypes, hence, impacting neurodevelopment [172]. Similarly, neuroinflammation induced by the activation of monocytes, macrophages, mast cells, and microglia, which can lead to ASD’s onset and development, may be modified epigenetically [173]. It was recently suggested that epigenetic modifications may be applied to reprogram Müller glia for retinal regeneration [174]. Interestingly, epigenetic influences may be responsible for accelerated biological aging of glial cells of patients with multiple sclerosis [175]. Finally, the potential impacts of the gut microbiome on the dysfunction of enteric and brain glia, as well as astrocytes, which, in turn, may affect neuronal functions in neurodevelopmental disorders including ASD should be considered [176] (Figure 2). In this regard, the impacts of gut microbiota disorders on the BBB and, indeed, on the pathology of ASD have been well documented [8,177,178,179,180]. Interestingly, high levels of p-cresol, which is produced by bacterial fermentation of protein in the human large intestine, and correlates with ASD severity, have been detected in blood, urine, and stool of children with ASD. These children also express other gut microbial metabolites, including SCFAs, free amino acids, indoles, and lipopolysaccharides, in their blood and urine [181]. Thus, it has been proposed that the analysis of gut microbes and the detection of microbial-derived metabolites in stool, blood, and urine may provide an alternative method for the early diagnosis of ASD [181].

Hence, VPA, APAP, and PPA by virtue of their interactions with glial cells, may also epigenetically affect the function of these cells. For example, VPA may stimulate the proliferation of glial precursors during cortical gliogenesis [114], and prenatal VPA may activate spinal microglia leading to allodynia, whereby pain is felt by a stimulus that does not normally provoke pain [182], or prenatal APAP’s detrimental effect on attention-related behavior, may be, at least partly, mediated via glial cells [183,184]. PPA interaction with glial cells and epigenetic modification of these cells has also been documented [153]. However, in these cases the exact epigenetic mechanism is yet to be elucidated.

### 5.4. VPA–Epigenetics–ASD

VPA, by inducing HDAC, interacts with histones, changes the DNA stereo arrangement by reducing its molecular order, and affects histones H1 and H3 conformations. It alters the expression of transcription factors, thus leading to the modulation of gene expression. All these epigenetic changes result in chromatin remodeling [185] (Figure 1).

### 5.5. APAP–Epigenetics–ASD

In contrast to VPA, the epigenetic effect of APAP is mediated via DNA methylation. This was demonstrated in 2017, whereby a prospective cohort study provided a potential causal link between APAP, neurodevelopmental disorders, and DNA methylation. APAP primarily affected the genes involved in oxidative stress, neural transmission, synaptogenesis, and olfactory sensory pathways [186]. It is noteworthy that altered olfactory response is evident in children with ASD and is more pronounced with increased autism severity. Indeed, it has been suggested that olfaction may provide a novel early nonverbal, non-task-dependent ASD marker, as well as serve in ASD intervention [187]. Moreover, recent investigations indicate that impairment in odor identification in ASD appears to be associated with mitochondrial dysfunction [188], another anomaly strongly linked to ASD [189] (Figure 1).

### 5.6. PPA–Epigenetics–ASD

As alluded to earlier, PPA disruption of biogenesis, bioenergetics, and metabolism in neurons and glial cells has made it a suitable compound in modeling ASD. It is now well recognized that the epigenetic effects of PPA, like APAP, are mediated via DNA methylation [182,183]. Specifically, it was shown that PPA attenuates IL-6 cytokine production through downregulation of the expression of the epigenetic modifier DNA methyltransferase 1 and inhibition of DNA methylation in both mice and humans [182,190]. Moreover, PPA modification of mitochondrial morphology and dynamics may, at least partially, be mediated via DNA methylation [183,184,191] (Figure 1).

## 6. Treatment Modalities for ASD

Although neither specific biomarkers nor cures for ASD are available, there are several ways to minimize symptoms and maximize abilities. In this regard, behavioral management therapy, cognitive behavioral therapy, speech language therapy, social skills training, and physical and nutritional therapies, as well treatments with medications for the alleviation of some symptoms, are common interventions [181]. The latter include the use of SSRI and tricyclic antidepressants, psychoactive or antipsychotic medications, stimulants, anxiolytics, and anticonvulsants [181,192]. Moreover, because oxidative stress, inflammation, and mitochondrial dysfunction are common pathophysiological mechanisms of ASD, drugs targeting these factors are being advocated [181]. In addition, probiotics, prebiotics, fecal microbiota transplantation, microbiota transfer therapy, antibiotics, and dietary adjustment methods may provide additional reliefs [181,192,193,194].

## 7. Implications for Novel Interventions in ASD

Thus, it is evident that different epigenetic mechanisms (e.g., deacetylation vs. methylation) may be mediating the action of the drugs implicated in ASD. This knowledge may lead to insights into targeted therapy via specific epigenetic manipulation to prevent or perhaps even reverse some of the detrimental consequences of ASD. Clearly, more work in this area is necessary. Nonetheless, hints of such interventions are already evident. For example, maternal treatment with butyrate, an HDAC inhibitor, was shown to ameliorate ASD-like traits in a mouse model of ASD [193,195]. Moreover, the behavioral effects were accompanied by neurochemical observations where cerebellar cortex hypertrophy, as well as Purkinje cell firing, and long-term synaptic plasticity deficits were prevented by butyrate [195]. A recent study suggests that butyrate supplementation could be a possible therapeutic intervention for lead-induced neurotoxicity [196]. Curiously, a strong association between lead and ASD has also been indicated [8]. Thus, lead-induced ASD-like symptoms may also be targeted by butyrate. Another HDAC inhibitor, vorinostat, ameliorated impairments in sociability and cognitive memory in an Ash1L-deletion-induced ASD/ID mouse model [197]. In addition to butyrate and/other HDAC inhibitors, studies are increasingly pointing to the potential utility of probiotics or, in some instances, psychobiotics, a combination of bee pollen and probiotics in ASD [198]. Moreover, dietary therapies composed of prebiotics (artichoke and Luteolin) and probiotics (yogurt and *Lacticaseibacillus rhamnosus* GG) led to improvements in oxidative stress and neuroinflammation, biochemical characteristics of ASD, in a rat model of ASD [199]. Hence, combination therapies aimed at correcting epigenetic influences, as well as use of probiotics, may offer novel interventions in ASD.

Reversing or inhibiting DNA methylation, which may have as much of a contribution to ASD as HDAC, could provide further potential venues to prevent or treat ASD. Although currently, pharmacological tools for such interventions are not available, potential manipulation of this epigenetic mechanism via diet has been proposed [200]. Indeed, the therapeutic potential of DNA hypomethylation in ameliorating erosive inflammatory arthritis has recently been indicated [201]. Thus, as our knowledge of this epigenetic mechanism and its potential manipulation progresses, therapeutic interventions in neurodegenerative–neuropsychiatric and neurodevelopmental disorders including ASD become tangible.

## 8. Conclusions

ASD is a serious neurodevelopmental disorder involving both neuronal and glial cells in the CNS. Maternal usage of certain drugs (e.g., VPA and APAP) during pregnancy and the early postnatal period may increase the risk of ASD in the offspring. Use of both drugs simultaneously is particularly worrisome, because they would influence both major epigenetic control mechanisms, DNA methylation and histone acetylation, at the same time, which could theoretically cause larger, synergistic disturbances in chromatin organization and gene expression.

Postnatal exposure to some chemicals such as PPA may have similar and potentially additive or compounding effects, further increasing ASD risk.

In addition, recent implications of the epigenetic influences on glial cells, as well as the involvement of the gut microbiota in the etiology of ASD, provide novel diagnostic and/or treatment targets. In this regard, hints of potential intervention in ASD via the manipulation of epigenetics, particularly via HDAC inhibition, as well as the use of pre- or probiotics, are already surfacing. It is anticipated that with further progress in our understanding of this phenomenon, more selective ways to prevent or treat ASD will become available.
